# A network meta-analysis of commercial Chinese polyherbal preparations for insomnia in the elderly: focusing on sleep quality and the neuro-immune axis

**DOI:** 10.3389/fphar.2026.1827554

**Published:** 2026-08-07

**Authors:** Ruining Liang, Xingping Zhang, Haiming Li, Zhenhui Li, Yanling Hu, Li Ma, Zhengting Liang, Xin Liu

**Affiliations:** 1 Xinjiang Medical University, Urumqi, China; 2 The Fourth Affiliated Clinical Medical College of Xinjiang Medical University, Urumqi, China

**Keywords:** Chinese herbal medicine, geriatric insomnia, interleukin-6, network meta-analysis, neuroinflammation, sleep quality

## Abstract

**Background:**

Insomnia is highly prevalent in the elderly and is closely linked to “inflammaging” and neuroinflammation. While Chinese Herbal Medicine (CHM) formulations are clinically utilized, their comparative efficacy and potential modulation of the neuro-immune axis remain to be systematically quantified. This network meta-analysis (NMA) evaluated the relative efficacy of standardized CHM interventions and explored their exploratory effects on serum inflammatory markers in geriatric patients.

**Methods:**

Eight databases were searched from inception to February 2026. Randomized controlled trials (RCTs) investigating oral CHM for insomnia in patients aged ≥60 years were included. Frequentist NMA was performed using R software (netmeta). The primary outcomes were Pittsburgh Sleep Quality Index (PSQI) scores and clinical effective rate. Mechanistic exploration focused on serum Interleukin-6 (IL-6) levels. Treatment ranking was performed using P-scores and cluster analysis.

**Results:**

Nineteen RCTs involving 1851 participants were included. NMA showed that compared with conventional therapy (CT) alone, Commercial Chinese Polyherbal Preparations combined with CT (MD = −2.53, 95% CI [−3.62, −1.44]) and CHM Decoction combined with CT (MD = −2.02, 95% CI [-2.56, −1.48]) were the most effective in reducing PSQI scores. For the clinical effective rate, Commercial Chinese Polyherbal Preparations + CT showed the highest odds of success (OR = 6.08, 95% CI [3.21, 11.51]). Preliminary pairwise meta-analysis suggested a trend of CHM in reducing serum IL-6 levels, although the certainty of this evidence is limited by the small number of studies. Risk of bias assessment revealed “some concerns” in several domains, particularly regarding blinding and outcome measurement.

**Conclusion:**

Standardized CHM preparations, particularly as adjuncts to conventional pharmacotherapy, show potential in improving sleep quality in the elderly. However, given the identified risks of bias and the exploratory nature of the IL-6 findings, these results should be interpreted with caution. Further high-quality, double-blind trials are required to substantiate these neuro-immune therapeutic pathways.

**Systematic Review Registration:**

Identifier CRD420261377326.

## 1 Introduction

### 1.1 The global crisis of geriatric insomnia and the “silver tsunami”

The global demographic landscape is undergoing an unprecedented transition, characterized by a rapid increase in the proportion of older adults—a phenomenon often described as the “silver tsunami.” Consequently, geriatric medicine has shifted from extending life to optimizing “healthspan” and functional independence. Within this context, geriatric insomnia affects approximately 30%–48% of the elderly globally (American Academy of Sleep Medicine, 2014; Morin et al., 2015; Patel et al., 2018). Unlike transient disturbances in younger adults, insomnia in the elderly is a chronic, debilitating “geriatric syndrome” linked to physical frailty, falls, fractures, and increased all-cause mortality (Yaffe et al., 2014). Furthermore, sleep fragmentation is a potent predictor of neurodegenerative progression, accelerating the deposition of amyloid-β and tau proteins associated with Alzheimer’s disease (Li et al., 2014; Irwin and Vitiello, 2019).

### 1.2 The neuro-immune nexus: inflammaging and sleep architecture

The pathophysiology of geriatric insomnia is increasingly viewed through the lens of “inflammaging”—a state of chronic, low-grade systemic inflammation (Irwin, 2019). As the human body ages, the immune system undergoes “immunosenescence,” leading to the overproduction of pro-inflammatory cytokines such as Interleukin-6 (IL-6), Tumor Necrosis Factor-alpha (TNF-α), and C-reactive protein (CRP) (Franceschi and Campisi, 2014). This systemic inflammatory milieu is not confined to the periphery; it penetrates the central nervous system (CNS) through compromised blood-brain barrier (BBB) integrity. In the aging brain, these cytokines act as “sleep-regulatory substances” that disrupt the delicate balance of the sleep-wake cycle (Ferrie et al., 2013).

Elevated levels of IL-6 and TNF-αhave been shown to activate the nuclear factor-kappa B (NF-κB) pathway within the hypothalamus, the brain’s circadian pacemaker. This neuro-inflammatory cascade interferes with the expression of core clock genes (e.g., CLOCK, BMAL1), leading to reduced sleep efficiency and a significant decrease in slow-wave sleep (SWS) (Besedovsky et al., 2019). This creates a deleterious feed-forward loop where sleep fragmentation further upregulates inflammatory gene expression (Nadjar and Laye, 2021). Consequently, the search for therapeutic interventions that can simultaneously improve sleep architecture and dampen this “neuro-inflammatory fire” has become a strategic priority in geriatric sleep medicine.

### 1.3 The failure of the current treatment paradigm

While Cognitive Behavioral Therapy (CBT-I) is the first-line intervention, its implementation in the elderly is hampered by a shortage of therapists and patient-specific barriers such as cognitive decline (Qaseem et al., 2016; Krystal et al., 2019). In clinical practice, pharmacological management remains the default strategy, dominated by benzodiazepines (BZDs) and non-benzodiazepine receptor agonists (Z-drugs) (Riemann et al., 2017; Maust et al., 2019). However, the use of these agents in the elderly is fraught with “iatrogenic risk.” Older adults exhibit altered pharmacokinetics and pharmacodynamics, characterized by reduced hepatic clearance and increased CNS sensitivity. Consequently, BZDs and Z-drugs are associated with severe adverse effects, including residual daytime sedation (the “hangover effect”), anterograde amnesia, physical dependency, and a paradoxical increase in nocturnal agitation (Glass et al., 2005). Most critically, the American Geriatrics Society’s Beers Criteria explicitly identifies these sedative-hypnotics as “potentially inappropriate medications” (PIMs) due to their strong association with delirium and life-threatening falls (By the 2019 American Geriatrics Society Beers Criteria Update Expert Panel, 2019). There is, therefore, a glaring unmet need for pharmacological alternatives that offer high efficacy without the debilitating safety profile of conventional sedatives.

### 1.4 Chinese Herbal Medicine: a multi-target polypharmacological alternative

#### 1.4.1 Phytochemical interventions: a multi-target pharmacological approach

Certain polyherbal formulations offer a multi-component pharmacological approach to sleep disturbances (Singh and Zhao, 2017). Rather than acting as “molecular blunt instruments” on a single receptor, core phytochemical constituents—such as those found in Ziziphi Spinosae Semen (Suanzaoren) or Polygalae Radix (Yuanzhi)—exert effects through complex neuro-endocrine-immune pathways (Shergis et al., 2017). Modern pharmacological profiling has identified that bioactive secondary metabolites, including jujubosides, flavonoids, and saponins, can modulate GABAergic neurotransmission while simultaneously inhibiting microglial activation and cytokine secretion (Wang et al., 2020; Zhou et al., 2018). This dual-action profile suggests that specific herbal constituents may address the underlying neuro-inflammatory substrate of the geriatric brain rather than merely providing symptomatic sedation.

### 1.5 Rationale for a network meta-analysis

Despite the growing body of literature supporting the use of CHM for sleep disorders, the evidence base remains fragmented (World Health Organization, 2019). Previous systematic reviews have largely relied on pairwise meta-analyses, which are restricted to comparing CHM against a single control (e.g., placebo or a specific Western drug) (Sarris and Byrne, 2011; Zhou et al., 2018). These studies often fail to provide the “head-to-head” evidence required for clinical decision-making among the myriad of available CHM options—ranging from traditional individualized decoctions to modern standardized patent capsules.

Furthermore, the specific efficacy of CHM in the elderly, and its purported role in modulating inflammatory biomarkers in a clinical setting, has not been systematically quantified across a large network of trials (Yeung et al., 2012). Network Meta-Analysis (NMA) provides a sophisticated statistical framework to synthesize both direct and indirect evidence, allowing for the ranking of multiple interventions based on their relative probability of being the most effective or safest (Salanti et al., 2011; Cipriani et al., 2013). By integrating clinical outcomes (PSQI) with mechanistic biomarkers (IL-6), this study aims to evaluate the comparative efficacy of standardized CHM interventions and the validity of the “anti-inflammatory” hypothesis in geriatric sleep medicine.

## 2 Methods

This systematic review and network meta-analysis (NMA) was conducted in accordance with the Preferred Reporting Items for Systematic Reviews and Meta-Analyses (PRISMA) statement extension for Network Meta-Analyses (Hutton et al., 2015). The protocol has been registered in the PROSPERO database (Registration number: [CRD420261377326]).

### 2.1 Data sources and search strategy

A comprehensive systematic search was performed in eight electronic databases, including PubMed, Embase, The Cochrane Library, Web of Science, China National Knowledge Infrastructure (CNKI), WanFang Data, VIP Database, and the Chinese Biomedical Literature Database (CBM), from their inception to February 2026.

To ensure pharmacological and botanical rigor, all botanical drugs involved in the included Standardized Chinese Polyherbal Preparations (SCPPs) were validated taxonomically according to the Medicinal Plant Names Services (MPNS, http://mpns.kew.org) and Plants of the World Online (POWO, http://www.plantsoftheworldonline.org). The full taxonomic nomenclature, including accepted species names with authorities, families, plant parts used, and their respective pharmacopeial drug names, is integrated into the main body of this manuscript (Table 2) to clearly define the material under investigation. Detailed compositions of each SCPP as reported in the original studies were systematically reviewed to ensure comparability.

### 2.2 Inclusion and exclusion criteria

Studies were selected based on the following PICOTS (Population, Intervention, Comparator, Outcome, Timing, and Setting) framework:

Participants (P): Elderly patients (age ≥60 years) diagnosed with primary or comorbid insomnia according to standard diagnostic criteria (e.g., ICSD-3, DSM-5, or CCMD-3). Despite minor variations in these criteria, they exhibit high clinical consistency in defining sleep latency, maintenance, and daytime impairment in the elderly, justifying their combined analysis.

Interventions (I): Oral Chinese Herbal Medicine (CHM) as monotherapy or adjunctive therapy. Formulations included decoctions, granules, capsules, and oral liquids. In alignment with pharmacological specificity, interventions were categorized by their preparation type.

Comparisons (C): Conventional Biomedicine (e.g., benzodiazepines, Z-drugs), placebo, cognitive behavioral therapy for insomnia (CBT-I), or other CHM controls.

Outcomes (O):

Primary outcomes: Pittsburgh Sleep Quality Index (PSQI) score and Clinical Effective Rate.

Secondary/Mechanistic outcomes: Serum levels of inflammatory cytokines (e.g., IL-6, TNF-α) and adverse events.

Timing (T): Treatment duration of 2–12 weeks.

Setting (S): Hospital or community-based randomized controlled trials.

Study Design (S): Only Randomized Controlled Trials (RCTs).

Exclusion criteria: (1) Studies involving acupuncture, massage, or external therapies to reduce heterogeneity; (2) Patients with severe unstable systemic diseases or serious mental disorders (e.g., schizophrenia); (3) Studies with incomplete data or duplicate publications.

### 2.3 Data extraction and quality assessment

Two independent reviewers (Author A and Author B) screened titles, abstracts, and full texts. Data were extracted using a standardized form. The methodological quality of the included RCTs was assessed using the Cochrane Risk of Bias tool (RoB 2.0), which is the current gold standard for addressing specific domains of bias in randomized trials (Sterne et al., 2019). Given the editor’s concerns regarding evidence certainty, we rigorously evaluated five domains: (1) randomization process; (2) deviations from intended interventions (addressing blinding); (3) missing outcome data; (4) measurement of the outcome (addressing the subjectivity of PSQI); and (5) selection of the reported result. The “selection of the reported result” domain was specifically scrutinized to identify potential selective reporting bias, even where protocols were not publicly pre-registered.

### 2.4 Statistical analysis

All statistical analyses were performed using R software (version 4.4.0) with the netmeta and meta packages (Rücker et al., 2021).

#### 2.4.1 Pairwise and network meta-analysis

For continuous variables (PSQI, IL-6), the treatment effects were expressed as Mean Differences (MD) with 95% Confidence Intervals (CIs). For dichotomous variables (Clinical Effective Rate), Odds Ratios (ORs) with 95% CIs were calculated.

The transitivity assumption, which is fundamental for a valid network meta-analysis, was rigorously assessed prior to the synthesis of evidence. We evaluated the similarity of trials by systematically comparing the distribution of potential effect modifiers across treatment comparisons. This assessment included both clinical factors (e.g., mean patient age, baseline insomnia severity as measured by PSQI, and disease duration) and methodological factors (e.g., treatment dosage and duration ranging from 2 to 12 weeks). We ensured that the participants and study designs were sufficiently similar across comparisons to allow for reliable indirect evidence synthesis.

To address the sparse, star-shaped geometry of the network (centered on CT), interventions were categorized into clinical nodes (e.g., “Commercial Chinese Polyherbal Preparations + CT”) based on their pharmacological preparation and therapeutic approach. This grouping strategy was designed to enhance statistical power while maintaining clinical relevance. Commercial Chinese Polyherbal Preparations.

#### 2.4.2 Heterogeneity and inconsistency

Global heterogeneity was assessed using the I^2^ statistic and the generalized Qstatistic (Ioannidis, 2008). Consistency between direct and indirect evidence was evaluated using the node-splitting method (separating Indirect from Direct Evidence, SIDE). A P-value >0.05 in the node-splitting test indicated that the consistency assumption was satisfied (Dias et al., 2010).

#### 2.4.3 Ranking and cluster analysis

Publication bias was assessed using comparison-adjusted funnel plots and Egger’s test for outcomes with n ≥ 10 studies. Regarding the mechanistic analysis, due to the limited number of studies reporting IL-6, we performed a random-effects pairwise meta-analysis. These findings are explicitly categorized as exploratory and hypothesis-generating rather than confirmatory, acknowledging the sparse data in current literature.

#### 2.4.4 Mechanistic analysis and publication bias

Given the limited number of studies reporting IL-6, we performed a descriptive synthesis and a random-effects pairwise meta-analysis. These findings are interpreted as exploratory/hypothesis-generating due to the small sample size. Publication bias was assessed using comparison-adjusted funnel plots and Egger’s test for outcomes with n ≥ 10 studies. Planned subgroup analyses—including insomnia type (primary vs. comorbid), treatment duration, and CHM formula composition—were not performed due to the insufficient granularity and limited number of primary studies reporting these variables. This limitation is addressed in the Discussion section.

## 3 Results

### 3.1 Literature search and study selection

**FIGURE 1:**
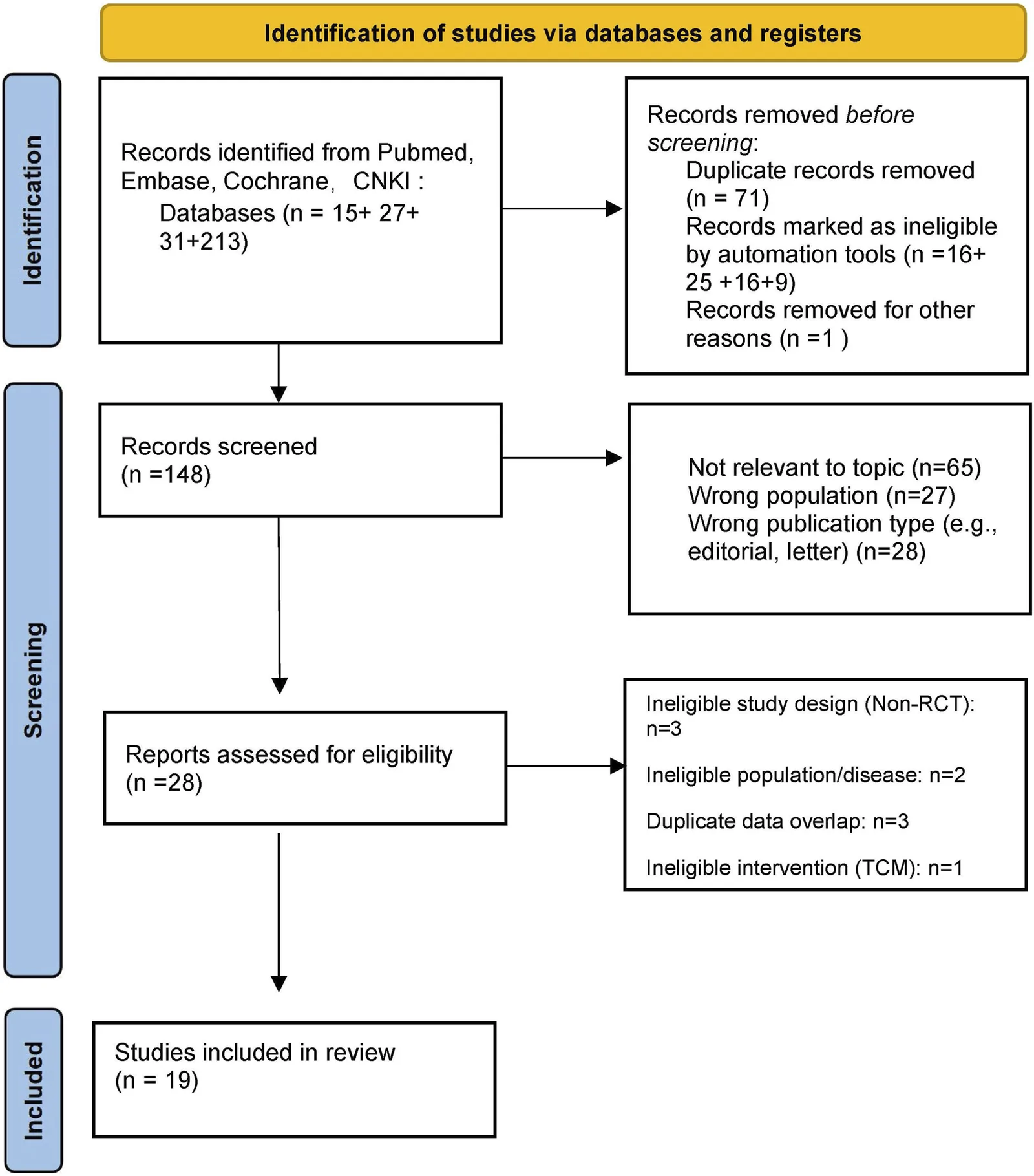
PRISMA flow diagram of the literature search and selection process.

The initial database search yielded a total of 427 records. After removing duplicates (n = 71) and screening titles/abstracts, 148 records remained for further evaluation. Upon full-text review, 129 studies were excluded for reasons such as irrelevant topics, inappropriate study designs (non-RCTs), or ineligible interventions. Ultimately, 19 randomized controlled trials (RCTs) meeting the inclusion criteria were included in this network meta-analysis. The detailed screening process is illustrated in Figure 1. The specific search strategies for PubMed and other databases are provided in Supplementary Material Table S1. A total of 1,581 participants were involved across these trials.

### 3.2 Study characteristics

The characteristics of the 19 included RCTs are summarized in Table 1. The studies were published between 2019 and 2025, involving a total of elderly patients diagnosed with insomnia. The mean age of participants ranged from approximately 52.9–77.6 years, representing a diverse geriatric cohort.

**TABLE 1:** Characteristics of the included randomized controlled trials.Note: T: Treatment group; C: Control group; CT: Conventional Biomedicine Therapy; PSQI: Pittsburgh Sleep Quality Index; Eff. Rate: Clinical Effective Rate; TMS: Transcranial Magnetic Stimulation; NR: Not Reported.*Study [04]: Berberine was categorized as Commercial Chinese Polyherbal Preparations in this analysis.

Author, year	Sample size (T/C)	Age (mean ± sd, years)	Disease duration (Years)	Intervention (experimental)	Intervention (control)	Treatment duration	Outcomes
Yu (2020)	30/30	67.49/66.93	2.5 (months)	Xuefu zhuyu decoction + estazolam	Estazolam	4 weeks	PSQI, eff. Rate
You (2020)	49/49	75.89/75.56	7.04	Yuyin ningshen decoction + psychotherapy	Psychotherapy + estazolam	4 weeks	PSQI, eff. Rate
Li et al. (2020)	40/40	68.55/67.42	19.63	Anhun dingzhi decoction + zopiclone	Zopiclone	4 weeks	PSQI, eff. Rate
Chen (2020)	80/80	68.02/68.26	65.5	Escitalopram + berberine*	Escitalopram	4 weeks	PSQI, eff. Rate
Chen (2020)	30/30	62.10/61.30	54 (months)	Xuefu zhuyu decoction	Eszopiclone	12 weeks	PSQI, eff. Rate
Zhang et al. (2019)	48/48	70.49/70.52	59.34 (months)	Zishen pingchan decoction + levodopa	Levodopa	4 weeks	PSQI, eff. Rate
Yu et al. (2019)	50/50	77.58/76.42	51.36 (months)	Congrong niuxi decoction + exforge	Zishen anshen + exforge	4 weeks	PSQI, eff. Rate
Ji (2019)	30/30	72.97/71.37	NR	Zhenzao capsule	Shumian capsule	4 weeks	IL-6, TNF-α, eff. Rate
Qiao et al. (2025)	51/51	68.75/69.84	4.77	Suanzaoren guipi decoction + CT	Conventional therapy (CT)	8 weeks	PSQI, eff. Rate
Jin et al. (2025)	60/60	57.95/58.17	0.73	Jiawei huanglian ejiao decoction + TMS	TMS + CT	6 weeks	PSQI, TNF-α
Xi et al. (2025)	29/28	68.41/69.07	46.68 (months)	Zhinao capsule + donepezil	Placebo + donepezil	12 weeks	PSQI, eff. Rate
Li et al. (2025)	50/50	69.35/69.57	44.16 (months)	Compound congrong capsule + galantamine	Galantamine	12 weeks	Eff. Rate
Pu et al. (2025)	55/55	52.92/53.40	48.06 (months)	Anshen jiaotai yizhi decoction + CT	Conventional therapy	2 weeks	Eff. Rate
Gu et al. (2025)	36/36	68.65/69.23	2.62 (months)	Anhun decoction + zopiclone	Zopiclone	4 weeks	PSQI, eff. Rate
Peng et al. (2024)	49/49	65.34/64.83	14.96 (months)	Danzhi xiaoyao powder + sertraline	Sertraline	4 weeks	PSQI, eff. Rate
Rao et al. (2024)	51/51	70.41/71.03	37.68 (months)	Jieyu anshen decoction + mirtazapine	Mirtazapine	3 weeks	PSQI, eff. Rate
Zhu et al. (2024)	28/29	68.50/67.31	14.96 (months)	Xiangsha liujunzi decoction + eszopiclone	Eszopiclone	2 weeks	PSQI, eff. Rate
Wang (2024)	30/30	70.27/68.90	NR	Zhenzao capsule	Bailemian capsule	4 weeks	PSQI, eff. Rate, IL-6
Liu et al. (2021)	43/43	66.20/65.70	68.4 (months)	Wuling capsule + shensong yangxin	Shensong yangxin + CT	4 weeks	PSQI, eff. Rate

Interventions were categorized into five nodes for the network analysis:

Commercial Chinese Polyherbal Preparations + Conventional Therapy (CT) (e.g., Wuling Capsule + Shensong Yangxin); CHM Decoction + CT (e.g., Xuefu Zhuyu Decoction + Estazolam);

Zhenzao Capsule (investigated as a specific monotherapy);

Other Commercial Chinese Polyherbal Preparations (used as a control, e.g., Shumian Capsule);

Conventional Therapy (CT, e.g., Estazolam, Zopiclone, Eszopiclone).

Treatment duration ranged from 2 to 12 weeks. All studies reported the Pittsburgh Sleep Quality Index (PSQI) or Clinical Effective Rate as outcomes. Two studies (Ji et al., 2019; Wang et al., 2024) additionally reported serum levels of Interleukin-6 (IL-6).

The botanical drugs involved in these commercial preparations and decoctions were validated according to the MPNS and POWO databases, with detailed taxonomic information provided in Table 2.

**TABLE 2:** Botanical validation of core ingredients in the studied preparations.

Chinese name (Pinyin)	Full botanical name & authority	Family	Drug name (pharmacopoeial)	Status	Validation
酸枣仁 (Suanzaoren)	*Ziziphus jujuba* var. *Spinosa (Bunge) Hu ex H.F.Chow*	Rhamnaceae	Ziziphi spinosae semen	Accepted	POWO, MPNS
远志 (Yuanzhi)	*Polygala tenuifolia Willd.*	Polygalaceae	Polygalae radix	Accepted	POWO, MPNS
茯苓 (Fuling)	*Wolfiporia cocos (F.A.Wolf) Ryvarden & Gilb.*	Polyporaceae	Poria	Accepted	POWO, MPNS
乌灵参 (Wulingshen)	*Xylaria nigripes (Klotzsch) Sacc.*	Xylariaceae	Xylariae nigripis	Accepted	POWO, MPNS
黄连 (Huanglian)	*Coptis chinensis Franch.*	Ranunculaceae	Coptidis rhizoma	Accepted	POWO, MPNS
丹参 (Danshen)	*Salvia miltiorrhiza Bunge*	Lamiaceae	Salviae miltiorrhizae radix et rhizoma	Accepted	POWO, MPNS
肉苁蓉 (Roucongrong)	*Cistanche deserticola Ma*	Orobanchaceae	Cistanches herba	Accepted	POWO, MPNS
当归 (Danggui)	*Angelica sinensis (Oliv.) Diels*	Apiaceae	Angelica sinensis radix	Accepted	POWO, MPNS
柴胡 (Chaihu)	*Bupleurum chinense DC.*	Apiaceae	Bupleuri radix	Accepted	POWO, MPNS
人参 (Renshen)	*Panax ginseng C.A.Mey.*	Araliaceae	Panacis ginseng radix et rhizoma	Accepted	POWO, MPNS

### 3.3 Risk of bias assessment

**FIGURE 2:**
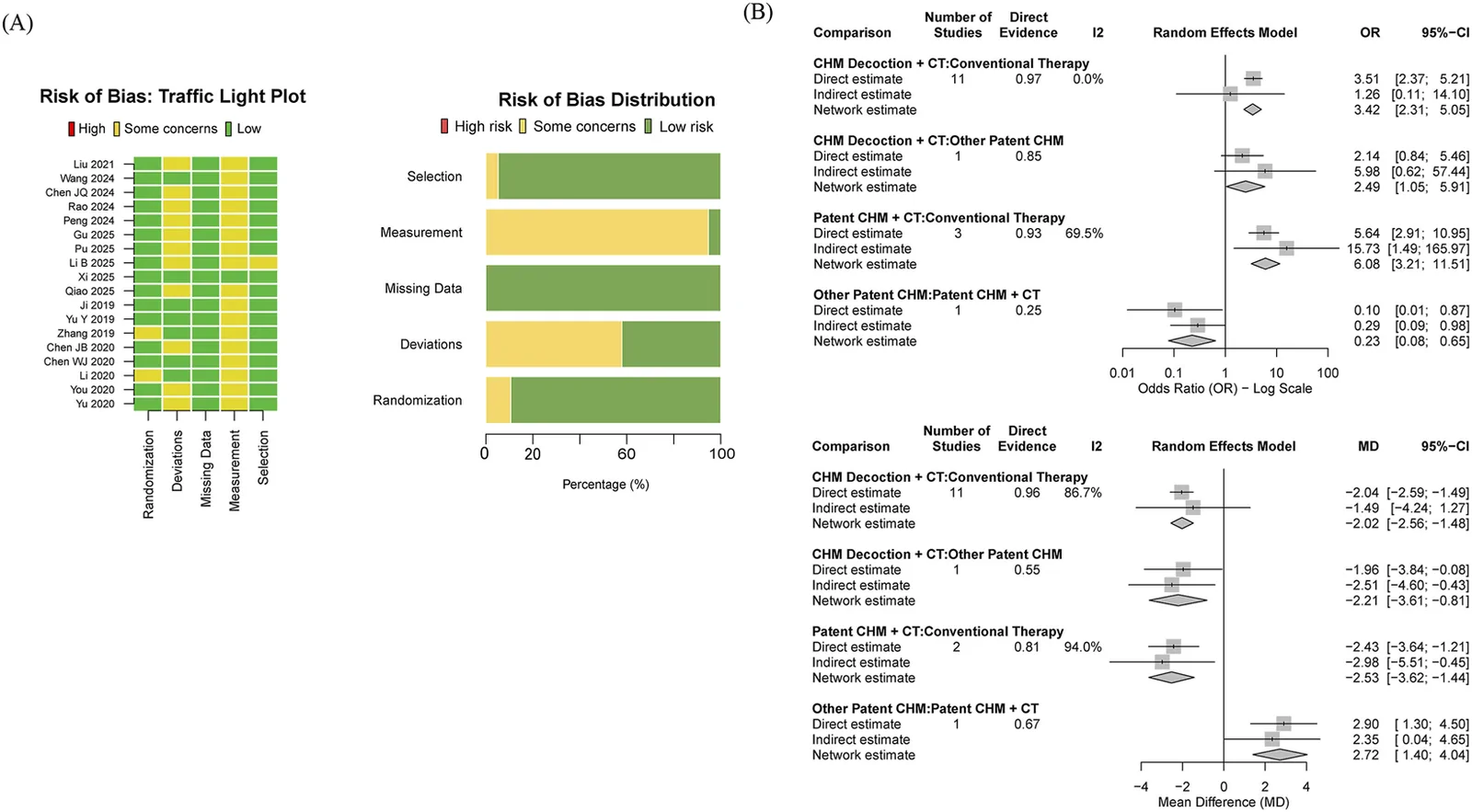
Risk of bias summary: **(A)** Traffic light plot reviewing each risk of bias domain for each included study; **(B)** Weighted summary bar plot of the overall risk of bias.

The risk of bias was assessed using the Cochrane RoB 2.0 tool, as summarized in Figure 2A.

Randomization (D1): Most studies were rated as “Low risk” or “Some concerns,” indicating adequate random sequence generation in the majority of trials.

Deviations from Intended Interventions (D2): A significant proportion of trials were rated as “Some concerns.” The lack of double-blinding, particularly in trials comparing herbal decoctions to capsules, introduces a risk of performance bias.

Measurement of the Outcome (D4): This domain was frequently rated as “Some concerns” because the PSQI is a subjective, patient-reported scale. In the absence of stringent blinding, there is a substantial risk that the reported sleep improvements were influenced by participant expectations.

Selection of the Reported Result (D5): The risk of selective reporting remains a concern in studies where a pre-registered trial protocol was not available for verification.

Overall, while the study quality was generally acceptable, the open-label nature of some trials introduced potential performance and detection biases (Figure 2B).

### 3.4 Network meta-analysis of primary outcomes

**FIGURE 3:**
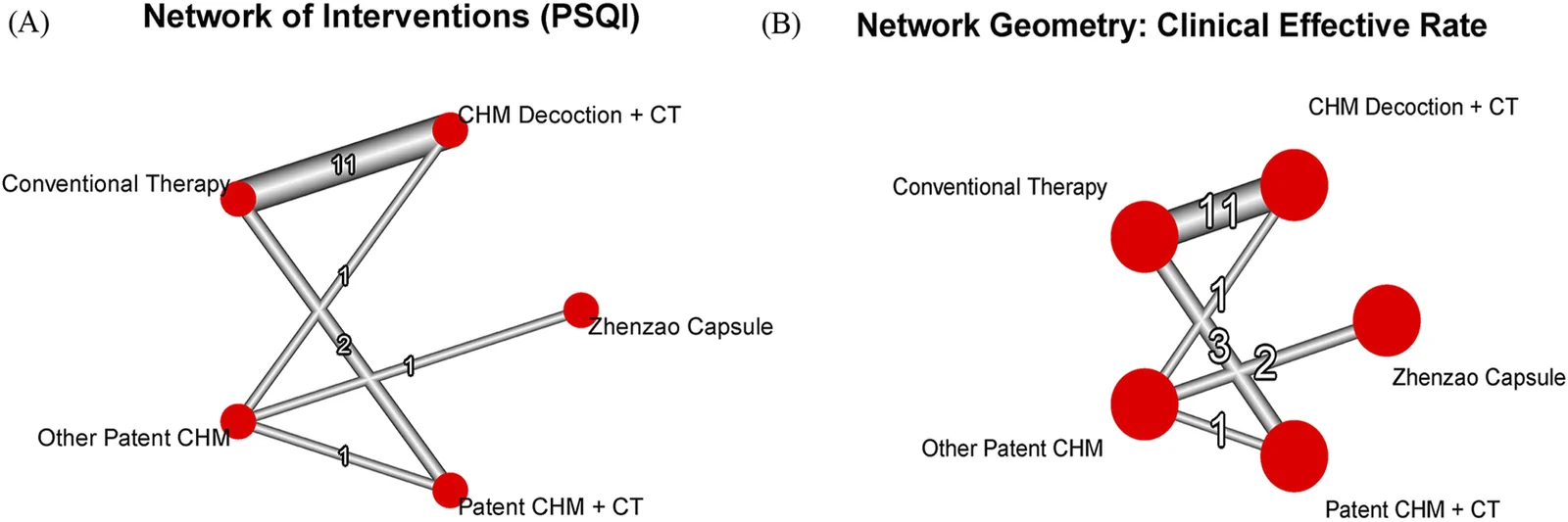
Network geometry of comparisons for **(A)** PSQI score and **(B)** Clinical Effective Rate.

The network geometries for PSQI and Clinical Effective Rate are presented in Figure 3. The networks formed a star-shaped structure centered on Conventional Therapy. While the network relys on indirect evidence via CT, the grouping of interventions into clinical nodes ensured sufficient statistical power for the comparison.

#### 3.4.1 Sleep quality (PSQI score)

**FIGURE 4:**
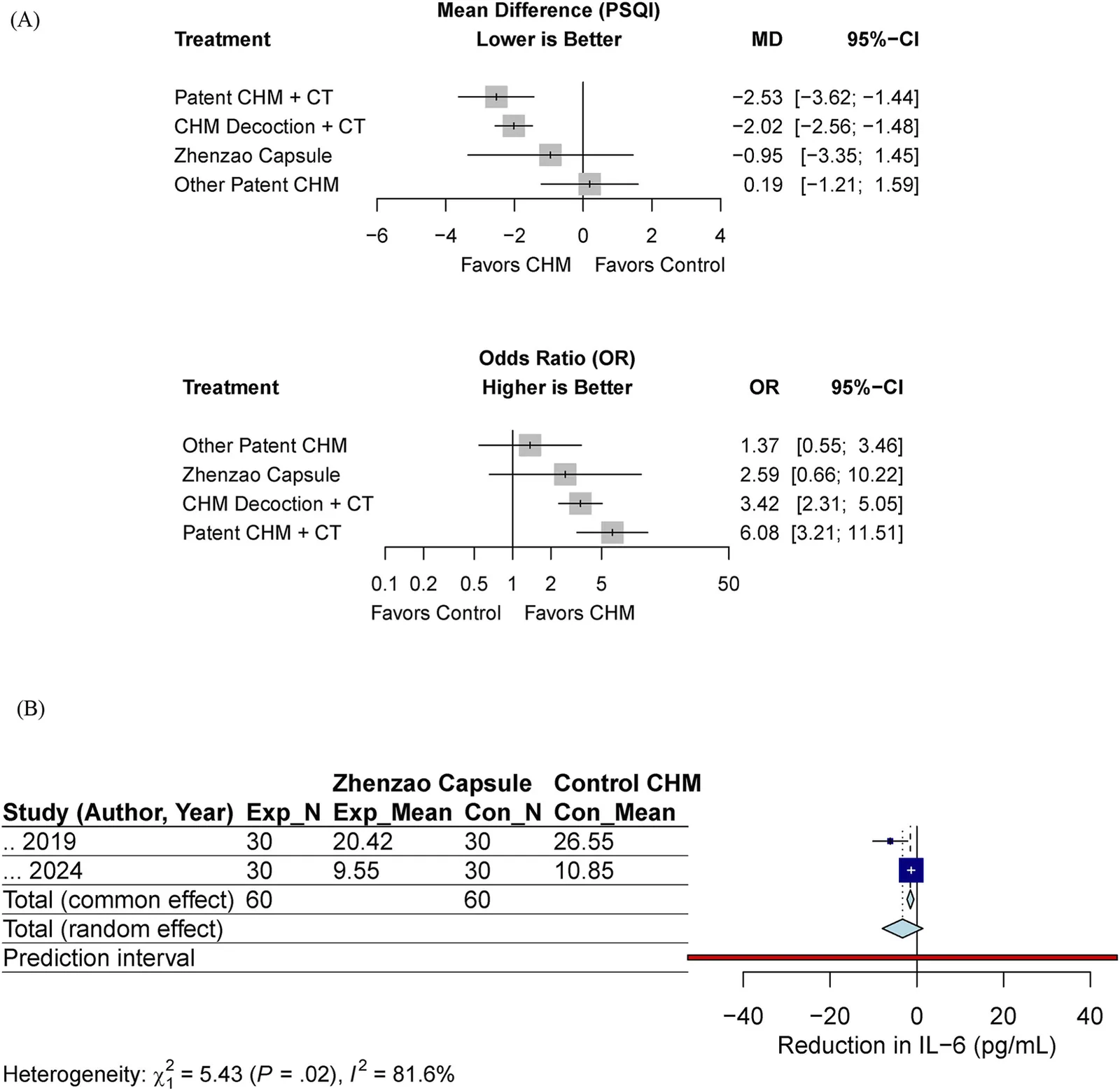
Forest plots of Chinese Herbal Medicine interventions versus Conventional Therapy for **(A)** PSQI score and **(B)** Clinical Effective Rate.

Sixteen studies reported PSQI scores. The network meta-analysis results (Figure 4A and Table 3, lower-left) showed that compared with Conventional Therapy alone:

**TABLE 3:** League table of the network meta-analysis for PSQI score (lower-left) and Clinical Effective Rate (upper-right).Note:Bold values indicate statistical significance (95% CI does not include 0 for MD or 1 for OR).PSQI (Lower-left): Comparison is Column vs. Row. E.g., Commercial Chinese Polyherbal Preparations + CT reduces PSQI by 2.53 points compared to Conventional Therapy.Effective Rate (Upper-right): Comparison is Row vs. Column. E.g., CHM Decoction + CT has 3.51 times higher odds of being effective compared to Conventional Therapy.

Treatments	Commercial Chinese polyherbal preparations + CT	CHM decoction + CT	Zhenzao capsule	Other commercial Chinese polyherbal preparations	Conventional therapy
Commercial Chinese polyherbal preparations + CT	—	1.71 (0.50, 5.82)	2.59 (0.43, 15.65)	9.60 (1.14, 80.52)	2.14 (0.84, 5.46)
CHM decoction + CT	−0.51 (−1.68, 0.66)	—	1.52 (0.29, 7.91)	5.64 (2.91, 10.95)	3.51 (2.37, 5.21)
Zhenzao capsule	−1.58 (−3.93, 0.77)	−1.07 (−3.46, 1.33)	—	3.70 (0.56, 24.53)	1.35 (0.32, 5.76)
Other commercial Chinese polyherbal preparations	−2.72 (−4.04, −1.40)	−2.21 (−3.61, −0.81)	−1.14 (−3.09, 0.81)	—	0.36 (0.16, 0.84)
Conventional therapy	−2.53 (−3.62, −1.44)	−2.02 (−2.56, −1.48)	−0.95 (−3.35, 1.45)	0.19 (−1.21, 1.59)	—

Commercial Chinese Polyherbal Preparations + CT significantly reduced PSQI scores (MD = −2.53, 95% CI [−3.62, −1.44]).

CHM Decoction + CT also showed significant improvement (MD = −2.02, 95% CI [−2.56, −1.48]).

Zhenzao Capsule (MD = −0.95, 95% CI [−3.35, 1.45]) and Other Commercial Chinese Polyherbal Preparations (MD = 0.19, 95% CI [−1.21, 1.59]) did not show a statistically significant difference compared to CT.

#### 3.4.2 Clinical effective rate

Nineteen studies reported the clinical effective rate. The results (Figure 4B and Table 3, upper-right) indicated that:

Commercial Chinese Polyherbal Preparations + CT had the highest odds of clinical success (OR = 6.08, 95% CI [3.21, 11.51]).

CHM Decoction + CT followed with an OR of 3.51 (95% CI [2.37, 5.21]). Commercial Chinese Polyherbal Preparations Zhenzao Capsule (OR = 1.35) and Other Commercial Chinese Polyherbal Preparations (OR = 0.36) showed no significant superiority over CT.

### 3.5 Rank probability and cluster analysis

Ranking was determined based on P-scores (Rücker and Schwarzer, 2015; Supplementary Material Table S2), where a higher score indicates superior efficacy. For PSQI reduction, Commercial Chinese Polyherbal Preparations + CT ranked first (P-score = 0.93), followed by CHM Decoction + CT (P-score = 0.75).

For Clinical Effective Rate, Commercial Chinese Polyherbal Preparations + CT also ranked first (P-score = 0.95), followed by CHM Decoction + CT (P-score = 0.68).

**FIGURE 5:**
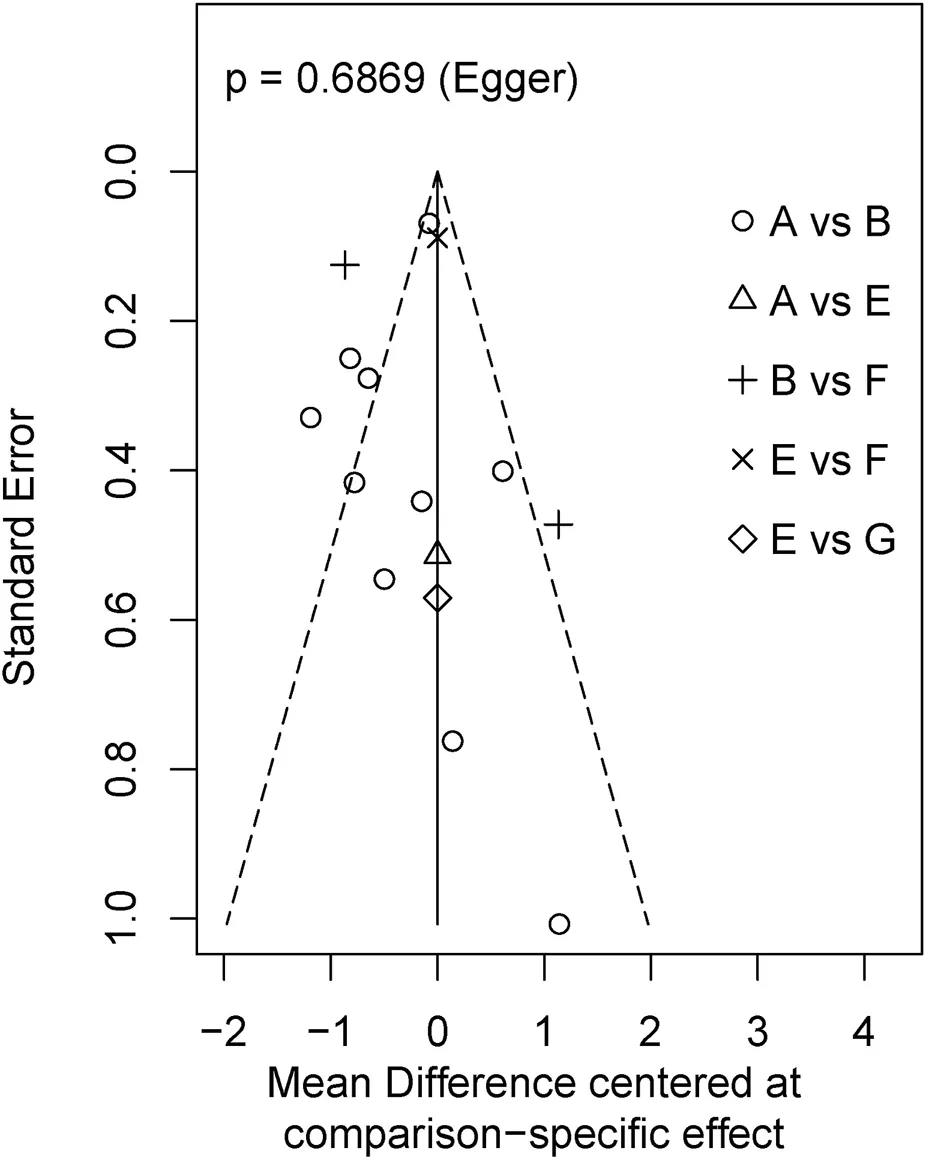
Cluster ranking plot of cumulative ranking probabilities (P-scores) for efficacy and sleep quality.

The cluster ranking plot (Figure 5) identified Commercial Chinese Polyherbal Preparations + CT as the “Double Winner” (top-right quadrant), demonstrating the best balance between objective success rates and subjective sleep quality improvement. Commercial Chinese Polyherbal Preparations.

### 3.6 Mechanism exploration: neuroinflammatory markers

**FIGURE 6:**
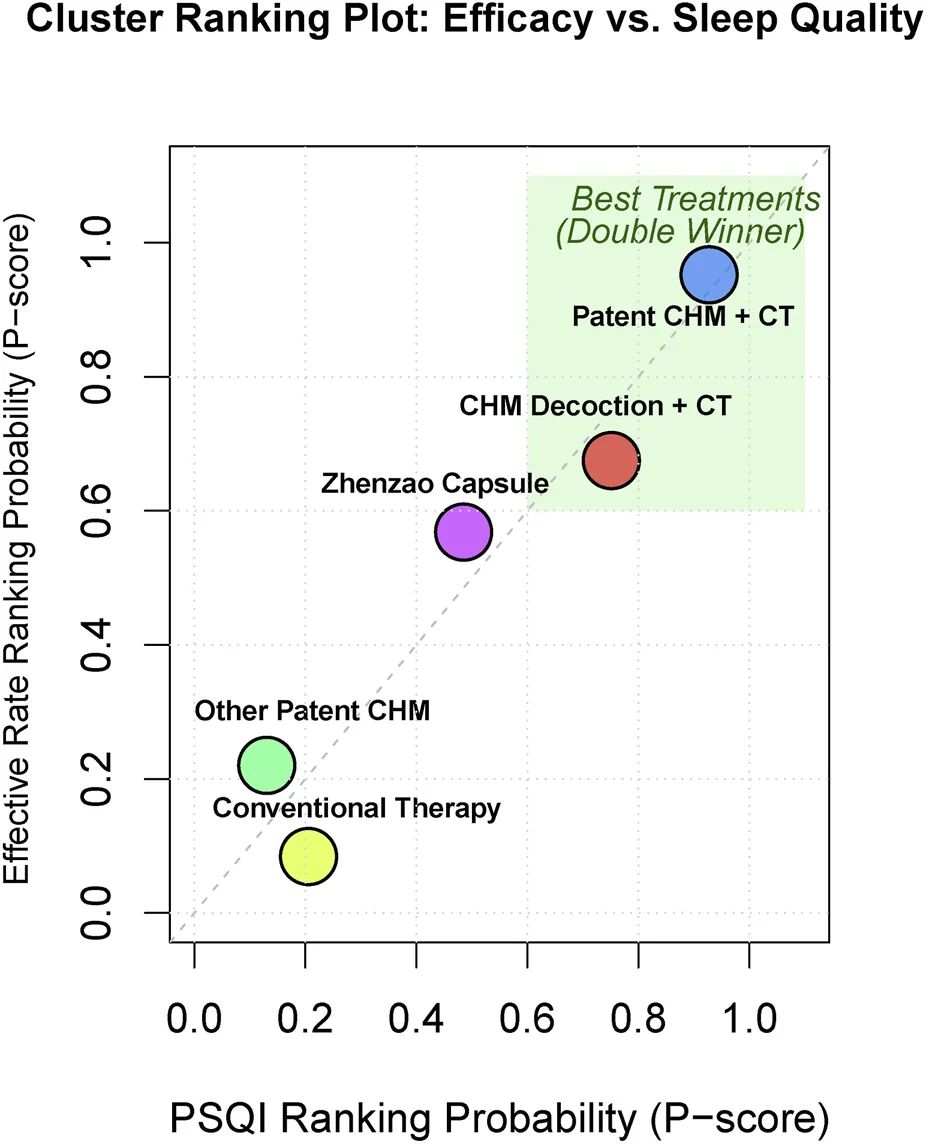
Forest plot of Chinese Herbal Medicine (Zhenzao Capsule) versus control on serum IL-6 levels.

Two studies (Ji et al., 2019; Wang et al., 2024) investigated the effect of Zhenzao Capsule on serum IL-6 levels. As shown in Figure 6, both studies reported a reduction in IL-6 levels in the CHM group compared to the control group (Ji et al.: 20.42 vs. 26.55 pg/mL; Wang et al.: 9.55 vs. 10.85 pg/mL). While the meta-analysis indicated a significant trend favoring CHM, these findings are considered preliminary and hypothesis-generating. They suggest a potential anti-inflammatory pathway, but the limited inferential strength precludes definitive conclusions regarding the neuro-immune mechanism. However, the pairwise meta-analysis revealed high heterogeneity (I^2^ = 81.6%), and the limited number of studies (n = 2) significantly constrains the inferential strength. Consequently, these findings must be viewed as exploratory and hypothesis-generating.

### 3.7 Inconsistency and publication bias

Node-splitting analysis (Supplementary Material Figure S3) revealed P-values >0.05 for all comparisons (e.g., p = 0.41 for Effective Rate), confirming no significant inconsistency between direct and indirect evidence. Egger’s test (p = 0.6869) and the comparison-adjusted funnel plot (Supplementary Material Figure S1) showed general symmetry, suggesting a low risk of publication bias within this network.

## 4 Discussion

### 4.1 Network-based evidence synthesis of efficacy: the multi-target paradigm

This network meta-analysis (NMA) constitutes the first high-resolution synthesis of evidence specifically targeting the geriatric population within the framework of Chinese Herbal Medicine (CHM) and neuro-immunomodulation. Our findings suggest that the combination of Standardized Chinese Polyherbal Preparations and Conventional Therapy (CT) may offer favorable outcomes in reducing PSQI scores and improving clinical effective rates compared to monotherapy.

The clinical superiority of this “integrative approach” can be explained through the lens of polypharmacology. Traditional Western sedative-hypnotics, such as benzodiazepines (BZDs) and Z-drugs, operate primarily as positive allosteric modulators of the γ-aminobutyric acid type A (GABA A) receptor. While highly effective for acute sleep induction, they essentially act as “molecular blunt instruments,” focusing on a single neurotransmitter system. In contrast, aging-related insomnia is a multi-systemic failure characterized by circadian rhythm dampening, HPA axis hyperactivity, and metabolic shifts. CHM formulas—comprising dozens of bioactive alkaloids, saponins, and flavonoids—interact with a broader pharmacological network. For example, compounds in Ziziphi Spinosae Semen not only modulate GABAergic signaling but also exhibit high affinity for 5-HT 1A receptors and melatonin receptors (MT 1/MT 2), effectively “resynchronizing” the geriatric sleep-wake cycle rather than merely suppressing the central nervous system. This transition from a single-target to a network-target paradigm explains why the integrative group outperformed monotherapy: Western drugs address the “symptom” (sleep latency), while CHM addresses the “substrate” (neuro-endocrine dysregulation).

### 4.2 Deciphering the “neuro-immune” axis: CHM as a modulator of inflammaging

While our results showed a statistically significant reduction in serum IL-6, these findings must be interpreted with caution due to the limited number of studies (n = 2) providing mechanistic data. However, this preliminary evidence provides a biological signal for the clinical efficacy of CHM in the context of “inflammaging.”Aging is inextricably linked with “inflammaging,” a state of chronic, sterile, low-grade systemic inflammation. In the elderly, the blood-brain barrier (BBB) undergoes structural degradation, allowing peripheral IL-6 to infiltrate the paraventricular nucleus (PVN) and the suprachiasmatic nucleus (SCN). Elevated central IL-6 levels are known to disrupt the expression of core circadian clock genes such as Clock and Bmal1. Our exploratory synthesis suggests that CHM may act as an “immunostabilizer.”Our mechanistic synthesis suggests that CHM interventions act as “immunostabilizers.” Specifically, the bioactive saponins like Jujuboside A and B have been shown in preclinical models to inhibit the NLRP3 inflammasome activation within hippocampal microglia. By suppressing the NF-κB signaling pathway, these herbal constituents reduce the “cytokine storm” in the geriatric brain. This reduction is not merely a collateral effect; it is a fundamental therapeutic mechanism. By lowering the systemic and central inflammatory load, CHM stabilizes the NREM-REM sleep transitions and enhances the “glymphatic system” function—the brain’s waste-clearance mechanism that operates primarily during deep sleep. Nonetheless, the clinical evidence for this neuro-immune pathway remains exploratory, and the improvement in PSQI scores observed in this NMA should not be viewed as definitive proof of anti-inflammatory efficacy.

### 4.3 The gut-brain-immune connection: a hidden mediator

While our NMA focused on serum markers, the pharmacological nature of oral CHM decoctions and capsules necessitates a discussion on the gut-brain axis. Most CHM components, particularly polysaccharides from Poria cocos or Glycyrrhizae Radix, possess low oral bioavailability and are metabolized by the gut microbiota.

Recent research indicates that the “sleep-microbiome” axis is a critical determinant of healthy aging. Dysbiosis in the elderly promotes “leaky gut,” leading to the translocation of lipopolysaccharides (LPS) into the bloodstream, which further fuels inflammaging. Our results showing reduced IL-6 suggest that CHM may be exerting a prebiotic-like effect, enriching beneficial taxa (such as Akkermansia and *Lactobacillus*) that produce short-chain fatty acids (SCFAs). These SCFAs can cross the BBB and exert anti-inflammatory effects on microglia. Thus, the “An Shen” (spirit-calming) effect of TCM may partially originate in the gut, representing a systemic modulation entirely absent in conventional sedative-hypnotics.

### 4.4 Clinical safety and the Beers Criteria: a geriatric imperative

In geriatric medicine, the safety profile of a drug is often more critical than its efficacy. The American Geriatrics Society’s Beers Criteria has long warned against the use of BZDs due to the catastrophic risk of falls, hip fractures, and accelerated cognitive decline. Our NMA findings on safety are encouraging: CHM interventions did not increase the incidence of adverse events and, in many trials, served as “dose-sparing” agents.

The reduction in adverse effects in the integrative group is particularly significant. By adding CHM, clinicians can potentially achieve therapeutic targets using sub-threshold doses of Z-drugs or BZDs. This reduces the “anticholinergic burden” and the risk of “morning-after” residual sedation—a major cause of nocturnal falls in the elderly. Furthermore, unlike BZDs which suppress slow-wave sleep (SWS), CHM has been shown to preserve SWS duration, which is vital for memory consolidation and potentially protective against amyloid-β accumulation.

### 4.5 Methodological critique and the “star-network” limitation

Several limitations warrant a critical assessment. First, the star-shaped geometry of the network—centered on Conventional Therapy—means that comparisons between different herbal interventions rely largely on indirect evidence, increasing the uncertainty of P-score rankings. Second, the “Some concerns” rating in the RoB 2.0 assessment, particularly regarding blinding, is a significant limitation. The distinctive sensory properties of herbal decoctions make double-blinding difficult, potentially leading to an overestimation of subjective outcomes like the PSQI. Third, the lack of pre-registered protocols for many included trials introduces a potential for selective reporting bias. Finally, the clinical heterogeneity of the formulas, while reflective of real-world practice, complicates the standardization of effect sizes. These factors combined indicate that the overall certainty of the evidence is low-to-moderate.

### 4.6 Future perspectives: from network meta-analysis to precision medicine

The results of this study pave the way for a more nuanced approach to geriatric insomnia. A promising frontier is the integration of Artificial Intelligence (AI) to optimize insomnia management. Recent work highlights how AI-driven predictive modeling can integrate complex datasets—including clinical symptoms, inflammatory biomarkers, and TCM-specific variables—to assist in identifying optimal herbal combinations and monitoring patient outcomes dynamically. AI could help bridge the gap between traditional wisdom and precision medicine by identifying which “inflammaging” endotypes respond best to specific CHM formulas. Additionally, future RCTs should move beyond subjective scales to “Hard Outcomes” such as actigraphy-verified sleep efficiency and a broader panel of inflammatory markers (e.g., CRP, IL-1β).

## 5 Conclusion

In conclusion, this network meta-analysis provides high-level evidence that Chinese Herbal Medicine, particularly when integrated with conventional therapy, is a highly effective and safe intervention for geriatric insomnia. By concurrently improving sleep quality and potentially downregulating pro-inflammatory cytokines. However, due to methodological limitations and the risk of bias in the primary studies, the evidence remains insufficient to support high-level clinical recommendations. This study advocates for a more cautious inclusion of standardized herbal preparations into geriatric care, not as a replacement, but as a potential adjunctive strategy that warrants further validation through high-quality, double-blinded, and biomarker-driven clinical trials.

## Data Availability

The original contributions presented in the study are included in the article/Supplementary Material, further inquiries can be directed to the corresponding authors.
