# Supplementary material for: A network meta-analysis of commercial Chinese polyherbal preparations for insomnia in the elderly: focusing on sleep quality and the neuro-immune axis
Source: Front Pharmacol. 2026 Aug 7;17:1827554. doi: 10.3389/fphar.2026.1827554 (PMC13493584; doi:10.3389/fphar.2026.1827554)
Supplement: Supplementary file 1 [file Supplementaryfile1.docx]

**Supplementary Materials**

| Search Set | | Search Terms |
| --- | --- | --- |
| #1 | Aged[Mesh] OR "Elderly"[Title/Abstract] OR "Older adults"[Title/Abstract] OR "Geriatric"[Title/Abstract] OR "Senior"[Title/Abstract] OR "60 years old"[Title/Abstract] | |
| #2 | Sleep Initiation and Maintenance Disorders[Mesh] OR "Insomnia"[Title/Abstract] OR "Sleep disorder*"[Title/Abstract] OR "Sleep disturbance*"[Title/Abstract] OR "Sleeplessness"[Title/Abstract] | |
| #3 | Medicine, Chinese Traditional[Mesh] OR "Drugs, Chinese Herbal"[Mesh] OR "Chinese Herbal Medicine"[Title/Abstract] OR "TCM"[Title/Abstract] OR "Herbal Medicine"[Title/Abstract] OR "Decoction"[Title/Abstract] OR "Capsule"[Title/Abstract] OR "Granule"[Title/Abstract] OR "Zhong Yi"[Title/Abstract] OR "Zhong Yao"[Title/Abstract] | |
| #4 | Randomized Controlled Trial[Publication Type] OR "Randomized"[Title/Abstract] OR "Placebo"[Title/Abstract] OR "Clinical Trial"[Title/Abstract] | |
| #5 | #1 AND #2 AND #3 AND #4 | |

Supplementary Table S1. Detailed search strategy for PubMed and other databases.Note: Similar strategies were adapted for Embase, Cochrane Library, and Chinese databases (CNKI, WanFang, VIP, CBM) using corresponding Chinese terms (e.g., "老年", "失眠", "中药", "随机对照").

| Rank | Treatment (Intervention) | P-score (PSQI Score) | P-score (Clinical Effective Rate) |
| --- | --- | --- | --- |
| 1 | Commercial Chinese Polyherbal Preparations + Conventional Therapy | 0.93 | 0.95 |
| 2 | CHM Decoction + Conventional Therapy | 0.75 | 0.68 |
| 3 | Zhenzao Capsule | 0.49 | 0.57 |
| 4 | Other Commercial Chinese Polyherbal Preparations | 0.13 | 0.22 |
| 5 | Conventional Therapy | 0.21 | 0.08 |

Supplementary Table S2. Ranking probability (P-scores) of all interventions.Note: P-score ranges from 0 to 1. A higher P-score indicates a higher probability of being the best treatment.

For PSQI, a higher P-score means greater reduction in sleep scores (Better sleep).

For Clinical Effective Rate, a higher P-score means higher odds of treatment success.

Commercial Chinese Polyherbal Preparations + Conventional Therapy ranked first in both outcomes.

Supplementary Figure S1. Funnel plots for the assessment of publication bias.

Supplementary Figure S2. Node-splitting analysis of inconsistency for (A) Clinical Effective Rate and (B) PSQI Score.
